# Peer Mentorship via Mobile Phones for Newly Diagnosed HIV-Positive Youths in Clinic Care in Khayelitsha, South Africa: Mixed Methods Study

**DOI:** 10.2196/14012

**Published:** 2019-12-10

**Authors:** Damian Hacking, Zodwa Mgengwana-Mbakaza, Tali Cassidy, Pumeza Runeyi, Laura Trivino Duran, Ruth Henwood Mathys, Andrew Boulle

**Affiliations:** 1 Medecins Sans Frontieres Operational Centre Brussels South African Mission Khayelitsha South Africa; 2 Centre for Infectious Diseases and Epidemiological Research University of Cape Town Cape Town South Africa

**Keywords:** youth, HIV, peer influence, retention in care, mobile phone, patient navigator

## Abstract

**Background:**

Youths in South Africa are poor utilizers of HIV health services. Medecins Sans Frontieres has been piloting youth-adapted services at a youth clinic in Khayelitsha, including a peer virtual mentorship program over mobile phones, piloted from March 2015 to May 2016.

**Objective:**

The objective of this study was to evaluate the effect of the peer mentorship program on youth engagement with HIV services and explore the acceptability of the program to both mentors and mentees.

**Methods:**

Antiretroviral initiation, retention in care (RIC), and viral load suppression were compared between youths engaged in the virtual mentorship program and two matched controls. In-depth interviews were also conducted for 5 mentors and 5 mentees to explore acceptability and impact of the program.

**Results:**

A total of 40 youths were recruited into the virtual mentorship program over the study period. Of these, data were obtained for 35 and 2 matched controls were randomly sampled for each. There was no difference in baseline demographics (eg, age, gender, and CD4 count). Mentees had increased antiretroviral initiation (28/35, 80% vs 30/70, 42% in matched controls) and viral load completion (28/35, 80% vs 32/70, 45%); however, no differences were found in viral load suppression or RIC at 6 or 12 months. Mentors reported being motivated to participate in the program because of previous personal struggles with HIV and a desire to help their peers. Mentees reported fears of disclosure and lack of acceptance of their status as barrier to accessing services, but they felt free to talk to their mentors, valued the mentorship program, and indicated a preference for phone calls.

**Conclusions:**

Peer mentorship in youths is acceptable to both mentors and mentees and appears to increase linkage to care and viral load completion rates.

## Introduction

### HIV in Youths in South Africa

There are an estimated 20 million youths aged 15 to 25 years in South Africa according to the 2011 census. In 2012, only 50% of the youths knew their HIV status [1]. Of the youths that test positive (estimated at 7%) [2], only 28% are accessing antiretroviral therapy (ART), 83% of which link to and are retained in care in the first 2 years and 81% of which achieve viral load suppression [3]. Adapting services to be youth-friendly overcomes many of the barriers with youths accessing care [4,5], and hence, in the urban informal settlement of Khayelitsha, Medecins Sans Frontieres (MSF) supports a stand-alone youth-only clinic, with youth-adapted services, including in-facility youth-friendly adherence clubs, point-of-care CD4 testing, and young counselors. This youth-friendly model of care has managed to achieve 50% ART initiation rates [6], an improvement on national figures, but still short of the World Health Organization’s 90-90-90 goals. Furthermore, many youths still start ART late in the course of HIV disease, even if their status is known, leading to a poorer outcome [7].

### Youths and the Role of HIV-Positive Peers

One measure that can increase linkage to care in newly diagnosed patients is to link them to peers living with HIV. The role of the peer is to act as a navigator through the health system, provide peer support and basic counseling, and visit the patient to encourage return to care [8]. Just 1 visit from a navigator can increase long-term linkage to care substantially [8]. Using navigators can be effective in youth-cohorts [9], not only for linkage to care but also for peer-to-peer support and health promotion [10]; however, there are high potential stigma and disclosure problems among youths (from family, friends, and the community), especially with home visits, as many youths have not disclosed their status to their family [11,12].

### Youths and Mobile Technology

The use of mobile technology for health-related functions by HIV-positive youths is increasingly common [13]. Primarily, mobile phones have been used to disseminate youth-adapted health information, link youths to health care services [14], and provide peer support networks. The mobile chat app platform Mixit has been used in South Africa for a variety of HIV- and health-related interventions targeting youths, from digital counseling support to health promotion [15]. The platform is principally chat room–based, with either public chat rooms or password-protected private ones, and also includes functionality such as quizzes and video content; however, since the introduction of more popular social media platforms such as Facebook, it has reduced in popularity substantially [16]. In South Africa, youths have expressed a desire to receive health information and health services via mobile technologies [15], and in 2012, MSF introduced mobile phone–based support groups (WhatsApp group chat rooms) ,to supplement existing in-person youth-adherence clubs , which were frequently used and valued by the youths [16].

### The Virtual Mentors Program

On the back of this success, a peer-to-peer mobile phone–based system, known as the Virtual Mentors program, was introduced in 2015 to link newly diagnosed youths with stable youths in care. The aim of this program was to determine if peer-to-peer mentorship, specifically between newly diagnosed HIV-positive youths and HIV-positive youths stable in care, could be successfully implemented using mobile phones as the primary means of communication. This virtual mentor approach catered more strongly toward the mobile phone–based online communication desires of the youths [17]. It also obviated the need for a physical home visit.

#### Recruitment and Role of Mentors

Virtual mentors were recruited annually from existing HIV youth-adherence clubs, on a voluntary basis, and underwent a 1-day training session conducted by MSF to capacitate them on their roles as navigators. Mentors could volunteer for more than 1 year, a minimum of 6 mentors were active at any given time, and a total of 19 mentors were used throughout the study. The virtual mentor would interact with the mentee via a mobile interface (SMS text messaging, call, or WhatsApp messenger) and was provided with 50 South Afican Rand (approximately US $3.50) per mentee to cover any data or airtime costs. The mentor was required to send 2 greeting messages to the mentee upon receiving their contact details. If the mentee did not respond after 3 days, the mentor then contacted the counselor to confirm the contact details of the mentee, and if these were correct, they informed the counselor that their mentee had not responded.

Once the mentor had made contact with the mentee, there were no stringent guidelines for their interaction (duration or content); however, mentors were provided with a messaging template (Multimedia Appendix 1) to guide them in their interactions with the mentee, which stipulated the minimum required engagement. The mentorship would conclude with the mentor inviting the mentee to attend their next HIV youth-adherence club. By visiting the mentor’s club, the mentee got an idea of what the youth-adherence club entails and would potentially be encouraged to join their own youth-adherence club. The youth-adherence club visit date also acted as a fixed date for the termination of the mentorship program, and as such, mentees could receive anywhere from 2 to 8 weeks of mentorship depending on their recruitment date, although continued communication beyond this point remained an option if the mentor so desired or if the counselor felt it necessary and requested it of the mentor.

#### Recruitment of Mentees

All patients who were aged between 12 and 25 years, who were newly diagnosed HIV positive at either clinic site, who at diagnosis either did not agree to start ART or were ineligible for treatment based on CD4 guidelines, and who declined to join an HIV youth-adherence club were eligible for the study. The counselor performing the HIV test offered the option of a peer mentor and would explain to the patient that the mentor was a young person also living with HIV who attended the clinic, would not come to their place of residence, and would not disclose their status. Consenting patients were added to a mentee sign-up sheet, which included their name, contact details, and preferred platform of communication (eg, WhatsApp and SMS). Mentees were then assigned to appropriate mentors by the youth-counselor coordinator, who attempted as much as possible to match mentors and mentees based on similar demographics or circumstances (eg, a pregnant mentee with a woman who had 1 or more children).

This paper investigates whether the virtual mentorship program was acceptable to youths, both mentors and mentees, through in-depth interviews and describes the mentees’ HIV- and ART-related outcomes in comparison with the matched controls.

## Methods

### Study Design

A mixed methods design was employed. A matched case-control quantitative component was used to assess if the virtual mentorship increased ART initiation of the newly diagnosed patients and positively affected their retention in care (RIC). An in-depth qualitative interview component was used to determine if the mobile phone mode of communication was a preferable means of peer support for the youths.

### Study Setting

The study was conducted at 2 stand-alone youth clinics in a high HIV prevalence context, the periurban informal settlement of Khayelitsha, Cape Town. The study was conducted from March 11, 2015, to May 24, 2016. This study was approved by the University of Cape Town’s Human Research Ethics Committee (Reference: 245/2016).

### Descriptive Data Collection and Analysis

Descriptive data for the virtual mentees cohort were collected from the Prehmis electronic medical record database, including demographic, headcount, and RIC statistics. Viral load data were collected through direct lookup of patient data via the National Health Laboratory Service’s online portal. Patient clinic folder audits were conducted for any missing data fields.

For each virtual mentee analyzed, we selected 2 matched controls who tested positive at the same facility. Mentees were matched to the 2 patients with the nearest HIV counseling and testing (HCT) dates, without replacement. All patients with missing HCT data were dropped. The outcomes measured are detailed in Table 1.

No statistical comparisons were calculated between the intervention group and the matched controls, which are presented as an approximate indication of the outcomes of youths in standard of care.

**Table 1:** Descriptive data outcomes measured in mentees and matched controls.

Outcome to be measured	Calculation
Linkage to ART^a^ care	Number of patients who tested positive and initiated ART/number of patients tested positive
Median time to linkage to ART care	Of those who initiated ART, difference between the date of HIV counseling and testing and the date of ART initiation
Median time out of care pre-ART	Of those who did not link within the first 6 months, total number of days late for pre-ART appointments
Retention in care on ART	Number of patients retained in care at 6 and 12 months/number of patients who initiated ART
Viral load completion	Number of viral load results available/number due for a viral load at 4 months
Viral load suppression	Number of patients with suppressed viral load (<400 copies/ml)/number of viral load results available

^a^ART: antiretroviral therapy.

### In-Depth Interviews and Analysis

Participants were selected for in-depth interviews based on their age and gender to get a representative sample of all the mentees and mentors recruited and to ensure that at least one male and one female and an older (>18 years) and a younger (<18 years) participant were included. The in-depth interviews followed the guide outlined in Multimedia Appendix 2. The in-depth interviews were held in a private room at the clinic, at a location of the mentee’s convenience, or telephonically. Written informed consent was obtained for all participants, or verbal informed consent was obtained in the case of telephonic interviews. Interviews were conducted in a mixture of English and Xhosa; however, audio transcripts of the interviews were transcribed into English by the interviewer before analysis. The in-depth interviews were analyzed through a grounded theory approach. Transcripts were coded for emergent themes by 2 researchers. Lists of the emergent themes were then compared and condensed into key themes.

## Results

### Descriptive Data

**Figure 1:**
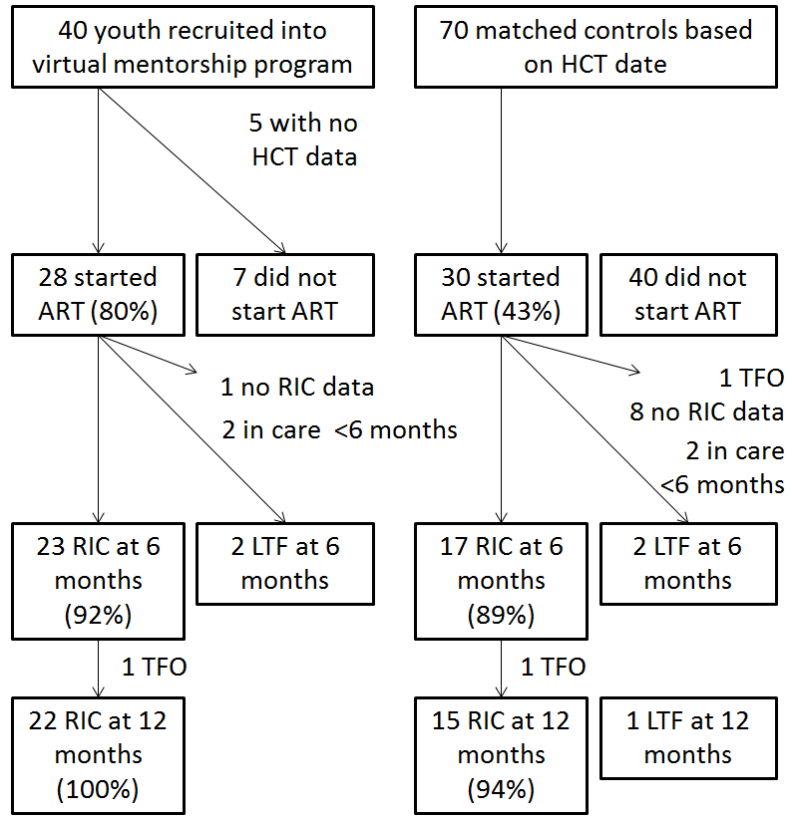
Exclusion criteria flow diagram. HCT: HIV counseling and testing; LTF: lost to follow-up; mo: months; RIC: retention in care; TFO: transferred out; ART: antiretroviral therapy.

A total of 40 youths were recruited into the Virtual Mentors program from March 2015 to May 2016. As detailed in Figure 1, 5 participants had missing HCT data and hence were excluded from analysis. Therefore, 70 matched controls with similar HCT dates were randomly drawn for comparison.

The mentees and the matched controls had similar demographic features and baseline CD4 counts, as detailed in Table 2.

**Figure 2:**
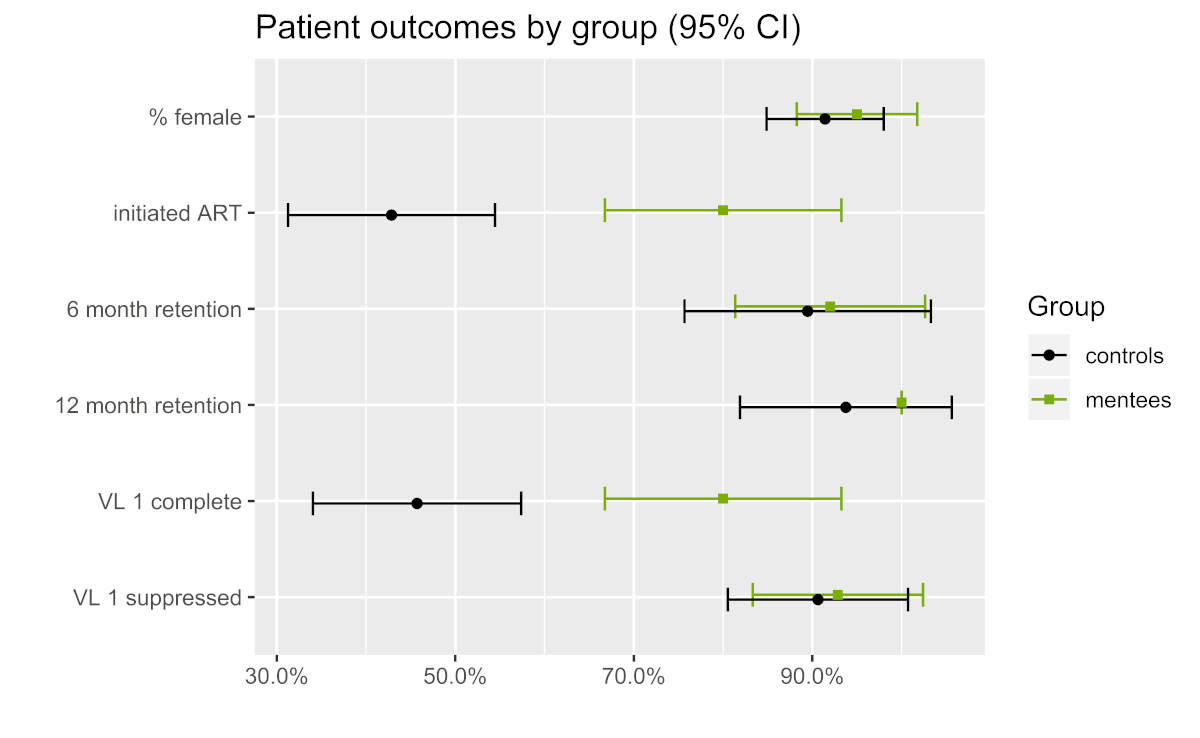
Antiviral therapy initiation, retention, and viral load completion by group. ART: antiretroviral therapy; VL 1: first viral load.

Linkage to ART care at any point after diagnosis was substantially higher in the virtual mentees cohort (Figure 2 and Table 3). Both groups had very large ranges in terms of the number of days between diagnosis and initiation of ART. RIC on ART at both 6 and 12 months was similar for both groups. Both groups had similar viral load suppression at the 4-month stage; however, the virtual mentees cohort had a much higher viral load completion rate.

**Table 2:** Demographic data between groups.

Demographic		Virtual mentees cohort (N=40)	Matched controls cohort (N=70)
**Gender, n (%)**			
	Male	2 (5)	6 (9)
	Female	38 (95)	64 (91)
Age at diagnosis, median (IQR^a^)		20 years 5 months (3 years 4 months)	22 years 7 months (3 years 6 months)
Baseline CD4, median (IQR); n		496 (378-592); 29	443 (307-638); 6

^a^IQR: interquartile range.

**Table 3:** Linkage, retention in care, and viral load outcomes.

Metric	Virtual mentors (VM)	Matched controls (MC)
Linkage to ART^a^ care, n (%)	28 (80)	30 (43)
Of those linked, Days to linkage to ART care, median (IQR^b^)	217.5 (32.5-467.75)	49.5 (7-333.25)
Days out of care pre-ART, median (IQR); n	38 (1-350); 17	200 (29-337.5); 11
Retention in care on ART at 6 months (for VM, N=45; for MC, N=19), n (%)	23 (92)	17 (89)
Retention in care on ART at 12 months (for VM, N=22; for MC, N=16), n (%)	22 (100)	15 (94)
First viral load completion (for VM, N=35; for MC, N=70), n (%)	28 (80)	32 (45)
First viral load suppression (<400 copies/ml; for VM, N=28; for MC, N=32), n (%)	26 (93)	29 (91)

^a^ART: antiretroviral therapy.

^b^IQR: interquartile range.

### Qualitative Data

#### Mentors

In total, 2 themes emerged from discussion with the mentors: (1) most mentors felt motivated to be a mentor because of previous personal struggles with their HIV status and (2) mentors saw the value in having a peer to speak to when newly diagnosed:

I decided to be a mentor because I was once a victim or was diagnosed with something and it was not easy for me to speak to anyone.

Participant 4

You see so I decided to take this and change other scared children you see to take their treatment and accept their status it is not easy to accept all of this you would think that you will die same time and how will you disclose at home you even need a person to speak to and you will not have so at least when you have someone your age who will speak the same language as you, you would feel better that is why I decided to take a step forward and be able to mentor other children.

Participant 1

#### Mentees

In total, 5 themes emerged from the interviews with the mentees: (1) a lack of acceptance acted as a barrier to care, (2) a fear of accidental disclosure to the community, (3) mentees felt free to talk openly with their mentors, (4) mentees had a positive view of the mentors asking them questions, and (5) direct phone calls and face-to-face interactions between mentors and mentees was higher than expected.

First, there were 2 major issues identified as barriers to care for youths: (1) lack of acceptance of their HIV status and (2) fear of disclosure if they attended the clinic or the support groups:

Problem I am scared of people they always check what you coming to do so I am avoiding that.

Participant 3

I was scared of here at Site C there can be someone from my area who will identify me know about my HIV, and go tell my parents about my involvement in the group I was scare to be known my HIV status and I had not yet accepted my HIV positive status so it was hard to attend support group I felt like hiding myself at home and be unknown.

Participant 2

The mentees had a very positive regard for the mentorship program and reported being able to talk to their mentors about disclosure issues and accepting their HIV status. The 2 major themes emerging were feeling free to talk openly to the mentor and a positive view of the mentors asking them questions:

She use to call me and ask things that I wished they were being asked by my mom or my older sister or something then I would speak with her and like I was free with her.

Participant 1

We were chatting nicely since she was a mentor who was supporting people who wanted people to accept their condition she was supportive towards people.

Participant 2

She was all right conversing encouraged me saying when you are HIV positive there are a lot of people who survive if they eat their treatment correctly but if you are not taking your treatment correctly HIV can kill you.

Participant 3

Finally, the primary modes of communication between mentors and mentees skewed toward direct phone calls and also included face-to-face interactions:

Yes I used to call her when there is something I do not understand.

Participant 3

She constantly phoned me and encouraged me to come to the group but I could not because I was stressed.

Participant 2

Ha a we used to meet when I am not fetching pills or coming to the club like when she asked to see me like if have come to fetch pills today she would say ok she will be free at a certain time and ask me to come tomorrow if there is nothing I am doing so we can talk nicely then I would come so that is how we were meeting.

Participant 5

## Discussion

### Principal Findings

#### Virtual Mentorship May Increase Antiretroviral Therapy Initiation and Mentees Have Good Retention in Care

Traditional adult patient navigator programs do not work well in the case of youths [9]. Our study shows that adopting the patient navigator role to be more youth friendly, that is, using peers and platforms acceptable to youths, is effective at linking youths to ART care. Youth-friendly peer navigators have been used successfully as part of the Red Carpet Program in Kenya [9] and the Health Connectors project currently underway in South Africa. The Health Connectors project also incorporates a mobile health (mHealth) element to their peer navigation [18]. The Virtual Mentors program described here differs from these interventions in that it comprises only of a peer mentor interaction over a mobile phone for a limited length of time. It is less resource intensive than either the Red Carpet Program or the Health Connectors project; however, it still had a positive impact, with substantially more youths linking to ART care than controls. In the virtual mentors group, there does appear to be a longer delay before linkage to care, and this could be a bias in the selection of mentees for the program or a result of the program itself. Of those who did not link within the first 6 months, the mentees were less likely to miss a pre-ART clinic visit.

#### Virtual Mentorship Is Acceptable to the Youth Mentors and Mentees and Counselors

The use of mHealth for youths in South Africa is well documented and has generally been well accepted across a variety of interventions [15]. The youths reported liking the virtual mentorship program, as they found the peers relatable and inspiring, which allowed them to freely talk to and ask questions of their mentors.

The mentors themselves were pleased to do the mentorship, and none of them reported it being an undue burden or that they felt out of their depth or unable to provide peer support to the newly diagnosed youths.

### Limitations

This study was limited by a small sample size. There were some challenges with sign up as only a small proportion of youths were signed up to the virtual mentorship program, given the large number of youths who were diagnosed positive at the clinic over the study period. It is unclear if these youths refused or if they were not offered the service.

The matched controls also displayed high levels of RIC, and these 2 factors made it difficult to elucidate any impact of the virtual mentors on long-term outcomes. Both the mentees and matched controls were also overwhelmingly female. This may limit the applicability of such a program to young males, a key population with poor access to HIV services; however, it is not indicative of a female preference for the program as the control group had a similar gender distribution.

Recruitment into the virtual mentees cohort was also at the discretion of both the counselor and the participant, and hence, there are likely strong selection biases at play. Counselors may have identified higher-risk individuals for recruitment into the program; conversely, participants with poor health-seeking behavior may have declined the services of a mentor. This may have biased both the virtual mentees cohort and the matched controls cohort (which either were not offered the service or refused). However, the lack of differences in baseline CD4 suggests that the youths accepting mentorship were at no higher or lower risk than the matched control.

There may have been a similar selection bias and recall bias for the in-depth interviews. Participants who defaulted treatment, or were not linked to care, were harder to contact and hence were underrepresented in the sample. So, it was difficult to understand the circumstances where the mentorship did not work.

### Conclusions

A peer-to-peer navigator program for newly diagnosed HIV-positive youths, conducted via mobile phone, might have increased linkage to ART care but had no impact on RIC. Mentees believed the program to be beneficial and supportive, and the mentors implementing the intervention were happy to do it.

### Implications for Policy and Practice

The benefit of community and peer health care workers in South Africa is well documented, and scale up of these services is a national policy [19]. Part of their role is to act as patient navigators [19-21]. There is also a greater recognition of the importance of adapting health care services to be youth friendly [22]. It is, therefore, vital that peer navigator services are also adapted to be youth-friendly. The virtual mentorship pilot presented in this paper has demonstrated a peer navigator approach, acceptable to both youth mentors and mentees, that could supplement or enhance any clinic wishing to implement youth-friendly services into their package of care.

## Appendix

Multimedia Appendix 1 Virtual mentors messaging template.

Multimedia Appendix 2 In-depth interview guide.

## Abbreviations

ART: antiretroviral therapy
HCT: HIV counseling and testing
mHealth: mobile health
MSF: Medecins Sans Frontieres
RIC: retention in care
