# Supplementary material for: Peer Mentorship via Mobile Phones for Newly Diagnosed HIV-Positive Youths in Clinic Care in Khayelitsha, South Africa: Mixed Methods Study
Source: J Med Internet Res. 2019 Dec 10;21(12):e14012. doi: 10.2196/14012 (PMC6930512; doi:10.2196/14012)
Supplement: Multimedia Appendix 1 [file jmir_v21i12e14012_app1.docx]

## Multimedia Appendix 1

**MESSAGING TEMPLATE**

**Introductory Session:**

**“Hi, I’m <name> from the virtual mentor program. Is this the right number?”**

**“I want to let you know that these messages will remain between the two of us only”**

Suggested Messages

*“how old are you?” / “I am XX years old” , “what do you do for fun?” / “I like to do …. For fun”*

*“Do you have any brothers or sisters?” / “My family consists of…” , “Do you have a boy/girlfriend?”*

**No Response AT ALL after 3 days**

**“Hi <name>, I haven’t heard from you. Are you there?”**

**Responded, but not again for 3 days**

**“Hi <name>, I haven’t heard from you in a while, is everything ok?”**

**Mentee initiates contact within 3 days**

**One week prior to Club Visit**

**“Hi <name>. I have my youth club visit on <date and time>. Have you heard about youth clubs?”**

**“Would you like to come with me to see what they are like?”**

Suggested Messages

*“The great things about youth clubs are it’s for youth like us, we can be ourselves and talk and laugh about things and share ideas, we are like a family. The things we talk about stay in the club.”*

**One day prior to Club Visit**

**“Hi, <name> don’t forget, we have our youth club visits at <time> tomorrow!”**

Suggested Messages

*“Let’s meet at…”*

*“Ask for Thembie, Charlien or Sonwabile when you get there”*

*“Let’s meet 5 minutes before the club starts?”*
