# Supplementary material for: Peer Mentorship via Mobile Phones for Newly Diagnosed HIV-Positive Youths in Clinic Care in Khayelitsha, South Africa: Mixed Methods Study
Source: J Med Internet Res. 2019 Dec 10;21(12):e14012. doi: 10.2196/14012 (PMC6930512; doi:10.2196/14012)
Supplement: Multimedia Appendix 2 [file jmir_v21i12e14012_app2.doc]

## In-depth Interview questions for Virtual Mentors and Mentees

**Mentors**

We are conducting a study to learn more about the experiences of mentors and mentees in the Virtual Mentorship programme, and would like to ask you a few questions. There are no right or wrong answers to the questions, and your decision to take part/not take part will not affect your involvement with the programme in any way.

1. Can you tell me why you decided to be a mentor?

**Probes:** Knowledge/interest in the programme, desire to help others, previous experiences of being a mentor/mentee

2. What did you think about the training you received before becoming a mentor? Was there anything in particular that you liked or didn’t like about the training?

3. What kind of support have you received during your time as a mentor?

**Probes:** support from other mentors, WhatsApp group support, support from counsellor, amount of support, suggestions for further support if feel needed

4. What kind of experience has being a mentor been for you?

**Probes:** positive or negative experience, time commitment, learning experience

5. Have you experienced any challenges during your time as a mentor?

**Probes:** Time commitment, amount of contact (too much or too little), problems with mentee

6. Understanding the relationship between mentor and mentees is key to evaluating the programme. Can you tell me more about your relationship with your mentee?

**Probes**: Role of gender and age, amount of support and interaction, experience of meeting up, any inappropriate contact or messages, problems with relationship, forming a friendship/relationship afterwards

7. Would you like to continue being a mentor? Can you tell me why/why not?

8. Is there anything you think we can do to improve in the programme in future?

**Probes:** Matching process, airtime or material reimbursement, training, support for mentors

Thank you for taking the time to talk to us today.

Do you have any questions you would like to ask us before we end the interview?

**Mentees**

We are conducting a study to learn more about the experiences of mentors and mentees in the Virtual Mentorship programme, and would like to ask you a few questions. There are no right or wrong answers to the questions, and your decision to take part/not take part will not affect your involvement with the programme in any way.

1. Can you explain to me what you understand by the Virtual Mentorship programme?

2. Why did you decide to take part in the programme?

**Probes:** support, didn’t feel ready to attend a youth club, choice

3. What kind of interactions have you had with your mentor?

**Probes:** SMS, WhatsApp, frequency and time of messages, experience of meeting(s) in person

4. Have you ever had any challenges with contacting your mentor?

**Probes:** Airtime, didn’t want people to know who they were messaging (disclosure), problems contacting them,

4. Did you go to a youth club with your mentor? If YES, can you tell me more about this meeting?

**Probes:** First impressions when meeting mentor, where they met, impressions of the group, discussion with mentor afterwards

5. If YES, do you think you will go to your own youth club? Can you explain why/why not?

**Probes:** disclosure, readiness,

7. Have you told anyone else about your involvement in the mentoring programme?

**Probes:** reasons, reaction of people, would they recommend it

8. Is there anything that you think we can do to improve the experience of mentees in the programme?

**Probes:** matching process, links to other mentees

Thank you for taking the time to talk to us today.

Do you have any questions you would like to ask us before we end the interview?
